# Structural Effects of Disease-Related Mutations in Actin-Binding Period 3 of Tropomyosin

**DOI:** 10.3390/molecules26226980

**Published:** 2021-11-19

**Authors:** Balaganesh Kuruba, Marta Kaczmarek, Małgorzata Kęsik-Brodacka, Magdalena Fojutowska, Małgorzata Śliwinska, Alla S. Kostyukova, Joanna Moraczewska

**Affiliations:** 1 Gene and Linda Voiland School of Chemical Engineering and Bioengineering, Washington State University, Pullman, WA 99163, USA; balaganesh.kuruba@wsu.edu (B.K.); alla.kostyukova@wsu.edu (A.S.K.); 2 Department of Biochemistry and Cell Biology, Faculty of Biological Sciences, Kazimierz Wielki University, 85-671 Bydgoszcz, Poland; marta92@ukw.edu.pl (M.K.); magdalena.fojutowska@ukw.edu.pl (M.F.); gosia.sl@ukw.edu.pl (M.Ś.); 3 National Medicines Institute, 00-725 Warsaw, Poland; m.kesik@nil.gov.pl

**Keywords:** tropomyosin, peptide, stability, congenital myopathy, cardiomyopathy, point mutations, coiled coil, circular dichroism, molecular dynamics simulation

## Abstract

Tropomyosin (Tpm) is an actin-binding coiled-coil protein. In muscle, it regulates contractions in a troponin/Ca^2+^-dependent manner and controls the thin filament lengths at the pointed end. Due to its size and periodic structure, it is difficult to observe small local structural changes in the coiled coil caused by disease-related mutations. In this study, we designed 97-residue peptides, Tpm1.1_64–154_ and Tpm3.12_65–155_, focusing on the actin-binding period 3 of two muscle isoforms. Using these peptides, we evaluated the effects of cardiomyopathy mutations: I92T and V95A in Tpm1.1, and congenital myopathy mutations R91P and R91C in Tpm3.12. We introduced a cysteine at the N-terminus of each fragment to promote the formation of the coiled-coil structure by disulfide bonds. Dimerization of the designed peptides was confirmed by gel electrophoresis in the presence and absence of dithiothreitol. Using circular dichroism, we showed that all mutations decreased coiled coil stability, with Tpm3.12_65–155_R91P and Tpm1.1_64–154_I92T having the most drastic effects. Our experiments also indicated that adding the N-terminal cysteine increased coiled coil stability demonstrating that our design can serve as an effective tool in studying the coiled-coil fragments of various proteins.

## 1. Introduction

Tropomyosin (Tpm) is a protein belonging to a family of actin regulatory proteins which bind to actin filaments to control interactions of actin with its binding partners. Two α-helical molecules of Tpm fold into a coiled coil and polymerize end-to-end to form uninterrupted chains along the entire length of actin filaments [1]. In response to fluctuations in sarcoplasmic Ca^2+^ concentration in striated muscles, Tpm together with troponin complex (Tn), regulates the actin-myosin interactions, and, thus, the contraction of muscle fibers [2]. In the contractile apparatus of different types of mammalian striated muscle, three isoforms of Tpm were found: Tpm1.1 expressed in cardiac and fast skeletal muscle, Tpm2.2 in cardiac, slow and fast muscle, and Tpm3.12 that is expressed exclusively in slow muscle fibers. The isoforms are products of *TPM1*, *TPM2*, and *TPM3* genes, respectively [3]. Although Tpm1.1 and Tpm3.12 are highly homologous proteins showing 91% identity and ~96% sequence similarity [4], their regulatory functions are quantitatively differentiated [5,6]. However, it is not known, which sequence differences are responsible for the regulatory performance of the isoforms. As a coiled-coil protein, the Tpm sequence is characterized by the presence of regular repeats of seven amino acid residues (labeled from *a* to *g*) known as heptad repeats. Hydrophobic amino acid residues, such as Val, Ile, Leu, are frequently present in the 1st (*a*) and 4th (*d*) positions of each repeat. Due to the α-helical fold of each Tpm chain, the hydrophobic residues are exposed on one face of the α-helix, which allows for locking them with the hydrophobic residues from the second chain in “knobs into holes” fashion. This forms a hydrophobic core necessary for dimerization and stabilization of two chains in the coiled-coil structure. Electrostatic, interhelical interactions between residues in *e* and *g* positions provide additional stabilization. In addition to the stabilizing hydrophobic residues, the core of Tpm coiled coil harbors clusters of small hydrophobic or polar residues such as alanine and serine, which cause bending and local flexibility thought to be necessary for Tpm to wrap around actin filaments [7,8].

The second periodic pattern along the Tpm sequence is related to its interactions with seven consecutive actin subunits. Each actin-binding period contains charged residues which are exposed on the outer surface of the coiled coil in *b*, *c*, and *f* positions of the heptad repeat [8]. Electrostatic interactions with oppositely charged residues on actin subunits mostly determine the interface between Tpm and actin [9,10]. Although the actin-binding periods are regarded as quasi-equivalent, they bind to actin with different affinities [11,12]. In addition to interactions with actin, the actin-binding periods have specific tasks, such as end-to-end interactions, binding TnT and TnI (the subunits of Tn complex), and interactions with the thin filament capping proteins and with myosin heads [13,14]. Several key structural features of Tpm periods are evolutionarily conserved and determine Tpm interactions with binding partners, stability, and flexibility [9,15,16,17,18,19].

A very conservative region of Tpm is the internal actin-binding period 3, the only period that is identical in the three striated muscle isoforms. A few amino acid differences between muscle isoforms are present in the close vicinity of period 3, but it is not known if these differences have any influence on period 3 structure. The deletion of the entire period 3 mildly impaired the binding of Tpm to F-actin in the presence of Tn (+Ca^2+^) [20], but severely affected cooperative allosteric changes in the filament that are important for interactions of myosin heads with the filament and development of isometric force [11,21]. The deletion of the Period 3 mutant drastically reduced the activation of the velocity of reconstituted thin filament movements on myosin head-covered surfaces [22]. This implies that structural changes within period 3 can be responsible for hypocontractile phenotype and muscle weakness. Nevertheless, point mutations found in humans in this region of Tpm are responsible not only for hypocontractile phenotypes related to reduced activation but also to hypercontractile phenotypes characterized by the increased activation of actin-activated myosin ATPase [23,24]. It is possible that disease-related mutations can change the stability and flexibility of the coiled coil which can manifest itself as a specific phenotype.

The aim of this work was to find out whether different phenotypes, caused by sequences of muscle isoforms and disease-related missense mutations in *TPM1* and *TPM3*, the genes encoding Tpm1.1 and Tpm3.12, can be associated with any particular structural changes in the mid-region of Tpm. To analyze local changes within period 3 and its vicinity, we constructed fragments of Tpm1.1 and Tpm3.12 containing the actin-binding period 3 and short sequences from periods 2 and 4. The effects of four autosomal dominant missense mutations found in human *TPM1* and *TPM3* on the structural stability of these fragments were analyzed by circular dichroism (CD) and molecular dynamic simulation (MDS).

## 2. Results

### 2.1. Design of Tpm1.1 and Tpm3.12 Peptides

**Figure 1:**
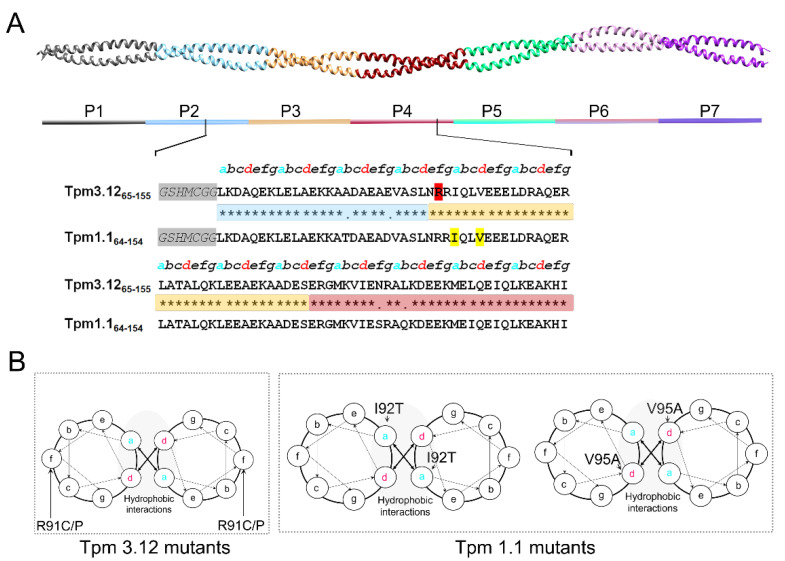
Tpm1.1 and Tpm3.12 peptides design. (**A**) Schematic representation of the fragments with respect to the full-length model of Tpm (PDB ID: 1C1G). Sequence comparison of designed Tpm3.12 and Tpm1.1 fragments. The alignment was performed in UniProt, accession numbers P09493 for Tpm1.1 and P06753 for Tpm3.12. Stars between the sequences indicate identical amino acid residues, dots indicate sites where the sequences differ. Blue, orange, and magenta indicate positions of actin-binding periods 2, 3 and 4, respectively. Lower case italicized letters on top of the sequences show positions of the amino acid residues in heptad repeat and *a* and *d* positions are shown in cyan and red, respectively. Mutation sites related to hypo- and hypercontractile phenotypes are highlighted in yellow and red, respectively. The N-terminal fusion peptide (shaded with gray) comprises amino acid residues left after proteolysis of His-tag with thrombin, followed by Cys and elastic Gly-Gly linker introduced during peptide construction. (**B**) Illustration of the coiled-coil wheel where positions of the mutations are indicated by arrowheads.

To limit the analyses to the conservative actin-binding period 3 and the sequences nearest to this period, we engineered 91-residue fragments of Tpm1.1 and Tpm3.12 comprising the entire 35-amino acid residue long actin-binding period 3 flanked by sequences of periods 2 and 4 at the N- and C-termini of period 3 (Figure 1).

To ensure the undisturbed folding of the coiled coil, the sequence contained 13 heptad repeats. The Tpm1.1 peptide contained sequences from Leu64 to Ileu154 and was referred to as Tpm1.1_64–154_. Since Tpm3.12 has an additional N-terminal Met, the amino acid residues in the sequence of Tpm3.12 have increased numbers compared to the sequence of Tpm1.1. For consistency with residue numbering in the full-length Tpm3.12, the peptide was designated as Tpm3.12_65–155_. The sequences of the peptides differed in the following residues: Thr79/Ala80, Asp84/Glu85 (actin-binding period 2), Ser132/Asn133, and Gln135/Leu136 (actin-binding period 4). In addition, the peptide carried an N-terminal fusion sequence with Cys residue separated from the Tpm sequence by a flexible Gly-Gly linker and GSHM tetrapeptide left after His-tag proteolysis (see Materials and Methods). The presence of Cys residues allowed us to cross-link two Tpm chains to facilitate the formation of the coiled coil structure.

The selected disease-causing mutations were located in a short segment of actin-binding period 3 (Figure 1). The substitutions I92T and V95A in Tpm1.1 are known to cause dilated cardiomyopathy and hypertrophic cardiomyopathy characterized by hypo- and hypercontractile phenotypes, respectively [24,25,26]. Substitutions R91C and R91P in Tpm3.12 are causative of congenital myopathy with slow muscle fiber hypotrophy and muscle weakness [23,27]. The cardiomyopathy mutations I92T and V95A were located in the *a* and *d* positions of the heptad repeat, in the core of Tpm1.1_64–154_ (Figure 1, Figure S1A). The congenital myopathy substitutions R91C and R91P were in the *f* position of the heptad repeat, which exposed them on the outer face of the Tpm3.12_65–155_ coiled coil (Figure 1, Figure S1B).

### 2.2. Structural Stability of Tpm1.1_64–154_ and Tpm3.12_65–155_

**Figure 2:**
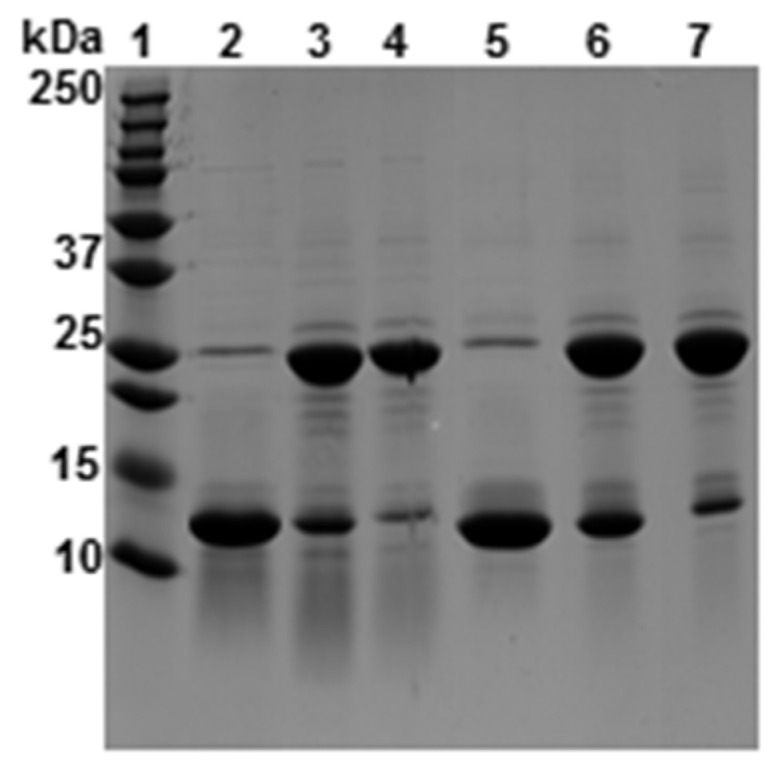
SDS-polyacrylamide gel electrophoresis of the wild-type peptides under reducing and non-reducing conditions. Tpm1.1_64–154_ (lanes 2–4) and Tpm3.12_65–155_ (lanes 5–7) separated on the SDS-gels after incubation for 3 min at 100 °C with Laemmli solution in the presence of 10% β-mercaptoethanol (lanes 2 and 5), in the absence of β-mercaptoethanol (lanes 3 and 6), or neither with boiling nor β-mercaptoethanol (lanes 4 and 7).

Structural analyses were performed under reducing and non-reducing conditions. To confirm the formation of Tpm cross-links via S-S bond formation, we used SDS gel electrophoresis. As shown in Figure 2, boiling the peptides in the presence of β-mercaptoethanol reduced the disulfide bonds and separated both chains of the dimers. The wild-type peptides Tpm1.1_64–154_ and Tpm3.12_65–155_, migrated with the apparent molecular mass ~11 kDa (lanes 2 and 5), which roughly corresponded to the theoretical molecular mass of one peptide chain. In the absence of β-mercaptoethanol, mobility of both peptides corresponded to the molecular mass of dimers ~22 kDa (lanes 4 and 7). The dimeric form dominated even after boiling the samples in the absence of β-mercaptoethanol prior to the separation on the gels (lines 3 and 6).

**Figure 3:**
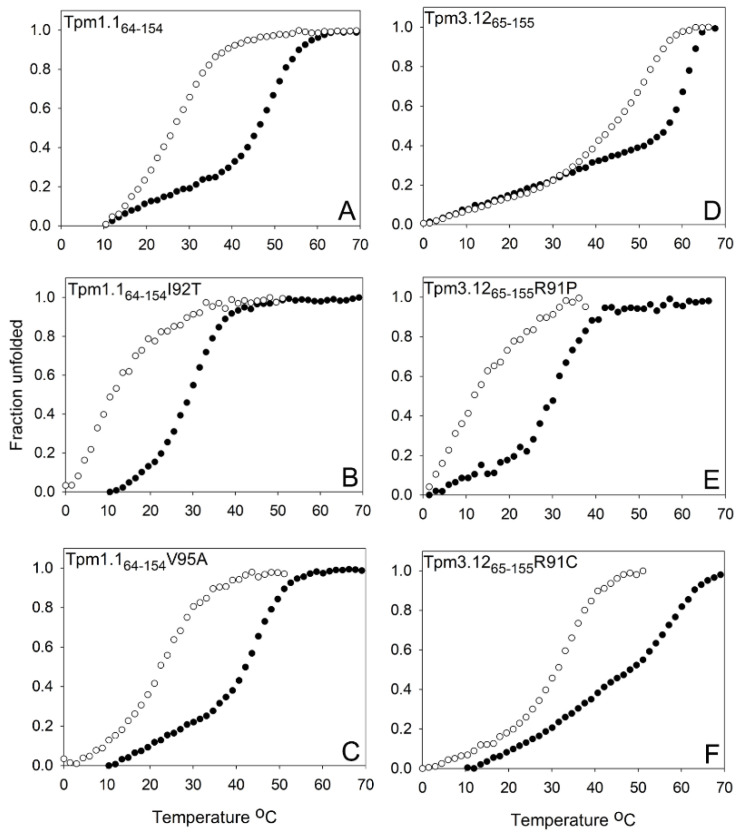
Effect of mutations on the thermal stability of Tpm1.1_64–154_ and Tpm3.12_65–155_. Melting of Tpm1.1_64–154_ (**A**), Tpm3.12_65–155_ (**D**), and their respective mutants (**B**,**C**,**E**,**F**) were measured at 222 nm in 100 mM NaCl, 10 mM Na-Phosphate, pH 7.0, with (○) and without (●) 0.5 mM DTT. Fraction unfolded is the ratio of unfolded to folded peptides (see Materials and Methods).

During the thermal unfolding experiments, a protein undergoes a transition from a folded to unfolded state due to a gradual increase in temperature. Considering the unfolding process undergoes a two-state transition (folded–unfolded), the temperature at which the ratio of folded to unfolded equals 1 is regarded as the melting temperature (T_m_). As T_m_ can be used to compare the structural stabilities of proteins with similar structures, the thermal unfolding experiments were performed under reducing and non-reducing conditions (Figure 3).

The values of T_m_ computed from the melting curves are collected in Table 1. The Tpm3.12_65–155_ cross-linked dimer displayed strong structural stability, which was approximately 15 °C higher than the cross-linked Tpm1.1_64–154_. Reducing the Cys residues with 0.5 mM DTT caused a significant drop in the stability of both peptides, but similarly to the oxidized peptides, the reduced Tpm3.12_65–155_ was more stable than the reduced Tpm 1.1_64–154_ (Table 1). While for Tpm3.12_65–155_ the difference in the T_m_ values between non-reduced and reduced species was approximately 15.1 °C, Tpm 1.1_64–154_ showed ~22.4 °C difference between reduced and non-reduced species.

### 2.3. Disruptive Effects of Missense Mutations on Stability of Tpm1.1_64–154_ and Tpm3.12_65–155_

In Tpm1.1_64–154_, the I92T mutation appeared to be more disruptive than the V95A mutation (Table 1, Figure 3). Under both reducing and non-reducing conditions, the I92T mutation resulted in a decrease in T_m_ by ~18 °C, and the decrease caused by the V95A mutation was only 3.9 and 2.0 °C for non-reduced and reduced peptides, respectively.

A decrease in stability was also observed in Tpm3.12_65–155_ when R91 was changed either to Cys or Pro. Under both non-reducing and reducing conditions, Tpm3.12_65–155_R91P was less stable than Tpm3.12_65–155_R91C. While the R91C mutation caused a ~9.4 °C decrease in T_m_, the R91P mutation resulted in a 32 °C decrease in T_m_ indicating a drastic decrease in thermal stability of the cross-linked Tpm3.12_65–155_. Under reducing conditions, T_m_ for Tpm3.12_65–155_R91P cannot be determined due to the lack of the beginning phase of the two-state transition (Figure 3E). In contrast, Tpm3.12_65–155_R91C showed the two-state transition (Figure 3F), as compared to Tpm3.12_65–155_, its T_m_ was decreased by ~14.5 °C.

### 2.4. R91P Mutation Increased Disorder in Tpm3.12_65–155_

**Figure 4:**
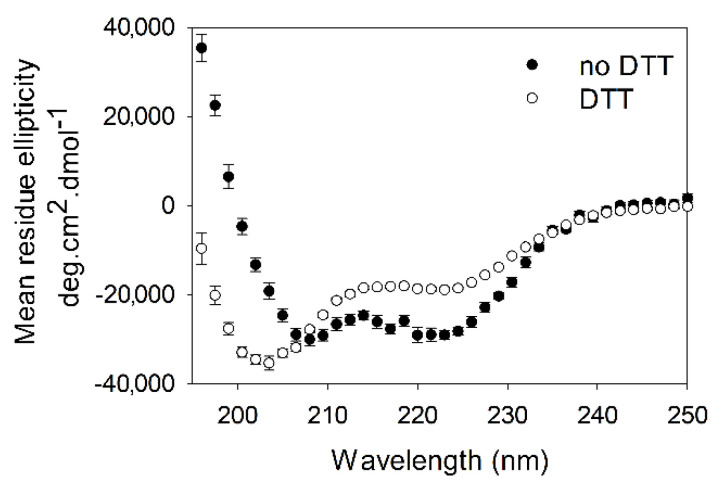
The R91P substitution in Tpm3.12_65–155_ results in decreased helicity in the presence of DTT. CD spectra were measured at 0 °C in 100 mM NaCl, 10 mM Na-Phosphate, pH 7.0, with or without 0.5 mM DTT.

In order to further evaluate the folding of Tpm3.12_65–155_R91P under reducing conditions, CD spectra were measured to evaluate the secondary structure changes induced by the R91P mutation (Figure 4). Under non-reducing conditions, Tpm3.12_65–155_R91P showed a profile characteristic for a protein that is mostly α-helical; however, upon treatment with DTT, the structure became significantly disordered as judged by the shift of the X-axis intersection point to lower wavelengths and the increase in the mean residue ellipticity at 222 nm from −28,370 to −19,057 deg.cm^2^.dmol^−1^, which accounts for the ~40% decrease in α–helical content.

### 2.5. Analysis of Three-Dimensional Structures of the Tpm Fragments Using Molecular Dynamics Simulations

No high-resolution 3D structures for Tpm1.1 and Tpm3.12 are available; therefore, we used a low-resolution (7 Å) X-ray crystal structure of cardiac α-tropomyosin from *Sus scrofa* (PDB ID 1C1G) as a template to create PDB files for Tpm1.1_64–154_ and Tpm3.12_65–155_. A Tpm fragment is an elongated molecule; therefore, end-to-end distance enables us to assess the bending propensity of this molecule. N and C termini of the designed fragments were disordered. To exclude the disordered regions and consider only the coiled-coil part of the Tpm fragment, Cα distances between the first and last residues forming the α-helix were measured over the course of the simulation. The Cα–Cα distances (Å) between L64–K152 in Tpm1.1_64–154_ (both chains), L65–E151, and L65–K153 in Tpm3.12_65–155_ (chains A and B, respectively), and their respective mutant fragments were measured and plotted as a function of time during the 200 ns simulation run (Figure S2, Table S1).

In Tpm1.1_64–154_, the range of end-to-end distance fluctuations was 55–95 Å (Figure S2). In Tpm3.12_65–155_, the range of fluctuations was 55–100 Å, which was similar to Tpm1.1_64–154_. Interestingly, the distances for the chains A and B in dimers fluctuated more independently in Tpm1.1_64–154_. Over the time of the simulation run, the distances were 78.24 ± 6.23 Å and 73.43 ± 5.74 Å for chains A and B, respectively (Table S1). In contrast, chains A and B of Tpm3.12_65–155_ maintained similar end-to-end distances with respect to each other (78.9 ± 9.3 Å and 76.7 Å ± 11.1 Å, respectively). The average distances for the fragments calculated as an average of the end-to-end distances of both A and B chains were 75.8 ± 4.9 Å for Tpm 1.1_64–154_ and 77.78 Å ± 9.9 Å for Tpm 3.12_65–155_. The differences in the average end-to-end distances for the individual chains can be attributed to the changes in the conformation of terminal residues (helical vs. extended).

We expected that introducing mutations in the pdb files of the fragments and running simulations would result in the disruption of the coiled-coil structures. Surprisingly, 200 ns simulation runs were not enough to show any effects on distances between individual chains of coiled coil. For all mutations besides I92T, there were also no significant changes in the end-to-end distances (Figure S2, Table S1). When the substitution I92T was introduced in Tpm1.1_64–154_, we observed fluctuations occurring between 40 and 80 Å, with an average distance 58.4 ± 7.2 Å.

To test if the distances measured during simulations were dependent on the positions of the side chains in the starting structures created in Chimera, we changed the orientation of the side chains of several residues that form intrachain salt bridges so that the salt bridges were broken. New chosen positions were also allowable and highly probable according to [28]. Then, simulations were repeated for Tpm1.1_64–154_ and Tpm1.1_64–154_I92T (Figure S3), and the average distances became 68.0 ± 6.8 and 75.73 ± 9.6, respectively. Based on this result, we concluded that the end-to-end distance was not a reliable parameter to determine the flexibility of coiled-coil structures created *in silico*.

## 3. Discussion

The flexibility of the Tpm coiled coil was reported to be crucial for the binding of Tpm to F-actin and the regulation of actomyosin interactions (reviewed by [1]). The highly conservative middle region comprising actin-binding period 3 is particularly important for the cooperativity of the actin filament regulation [20,21]. In humans, mutations in Tpm genes encoding actin-binding period 3 were linked to congenital myopathies and cardiomyopathies, muscle diseases that are manifested either by hypo- or hypercontractile phenotypes. In different Tpm genes, disease-causing mutations do not overlap, indicating that some regulatory properties are isoform-specific. It is not clear whether any particular structural changes in Tpm can be associated with Tpm functions leading to these phenotypes. To address this problem, in this work, we investigated the structural stability of peptides equivalent to sequences of the middle regions of Tpm1.1 and Tpm3.12, Tpm isoforms expressed in fast and slow muscle fibers, which contained sequences of actin-binding period 3 flanked by sequences from periods 2 and 4. The stabilities of wild-type peptides were compared to the stabilities of peptides carrying disease-causing missense mutations in period 3, which cause hypo- and hypercontractile phenotypes. CD measurements of thermal stability revealed that the middle region of Tpm3.12 was more stable than Tpm1.1. The mutations decreased thermal stability in the peptides with different degrees and had various effects on the peptide structure, but we did not observe any particular structural changes which determined the opposite phenotypes.

### 3.1. Differences in Stability of Tpm1.1 and Tpm3.12 Middle Regions

Thermodynamic studies of the stability of full-length Tpm1.1 and Tpm3.12 revealed the presence of three calorimetric domains in both isoforms, one of which was assigned to the N-terminal half of the molecule; for Tpm3.12, the thermal transition of the *N*-terminal domain was approximately 7 °C higher than for Tpm1.1 [29]. Although the peptides Tpm1.1_64–154_ and Tpm3.12_65–155_, which were studied in this work, contained only part of the *N*-terminal domain, they showed an even bigger, ~15 °C, difference in the thermal transition from fully folded to unfolded conformation. Tpm1.1_64–154_ was less stable than Tpm3.12_65–155_. The lower stability can be ascribed to the four amino acid differences between the peptides (Figure 1). It is interesting that the isoform-specific amino acid residues are located in the *b*, *g* and *f* positions of the heptad repeat. The *g* position amino acid residue, which electrostatically stabilizes the coiled coil, is a conservative Asp/Glu substitution; therefore, one can suppose that the high stability of Tpm3.12 mid-region is maintained by long-range conformational changes.

Because a certain degree of flexibility positively affects the binding of Tpm to actin, the difference between Tpm1.1_64–154_ and Tpm3.12_65–155_ observed in these studies may explain a higher affinity of Tpm3.12 for actin [5,6]. In agreement with the stabilizing effect of single Cys oxidation on the full-length Tpms [29], cross-linking of the peptide chains by oxidation of the N-terminal Cys increased T_m_. Together, the results obtained in this work indicate that the middle region extending from Leu64(65) to Ser154(155) significantly contributes to the conformation and stability of Tpm1.1 and Tpm3.12.

### 3.2. Effects of Disease-Causing Mutations on Structural Transitions of Tpm1.1 and Tpm3.12 Middle Regions

From the CD experiments, drastic decreases in the stability of the designed Tpm peptides in the presence of DTT indicate that the SS bond formed by cysteines in the N-terminal sequence *GSHMCGG* was effective in stabilizing Tpm’s coiled-coil structure. This addition to the N-terminal sequence could serve as an effective strategy in assessing the structural and functional relevance of Tpm’s periodicity in actin regulation and effects of other disease-related mutations in Tpms. The SS bond formed by the introduced N-terminal Cys mimics the stable N-terminal coiled coil that would precede this region in full-length Tpm and allows to ensure the formation of a coiled coil by the peptide. Introducing an additional C-terminal Cys that will form one more SS bond is not necessary for the coiled coil formation; furthermore, it may introduce an unnecessary limitation in observing the downstream structural effects of the mutations in the coiled coil. Due to the SS bond formation, our fragments have increased stability. For Tpm3.12_65–155_, T_m_ is 61 °C (cross-linked), while T_m_ of the corresponding region in full-length Tpm3.12 (Domain 3) is ~57 °C (reduced) or ~58 °C (cross-linked) [29,30]. For Tpm1.164–15, Tm is 46 °C (cross-linked), while T_m_ values of domains of full-length Tpm1.1 vary from 38 to 51 °C (reduced) [31]. Therefore, our designed cross-linked fragments have stability close to full-length Tpm.

As shown in Figure 1, Tpm 1.1 mutations I92T and V95A are in the core of the coiled coil (*a* and *d* positions). These mutations were shown to be associated with DCM and HCM respectively [24], indicating opposite effects of these mutations on the functions of Tpm. Tpm1.1_64–154_ V95A displayed structural stability similar to that of wild-type Tpm1.1_64–154_ as indicated by ~4 °C and ~2 °C decreases in the presence and absence of DTT (Table 1). Melting experiments indicated that Tpm1.1_64–154_ V95A performed better in the absence of DTT in retaining the coiled coil structure. In contrast to V95A, I92T mutation in Tpm1.1_64–154_ resulted in a significant decrease in the coiled coil stability, as suggested by the drop in T_m_ of ~18 °C in the presence and absence of DTT.

R91 in Tpm 3.12 makes an electrostatic interaction with actin, and it has been previously shown that substitution of R91 with Cys reduced Tpm’s actin affinity, thereby affecting F-actin pointed end regulation [23]. Based on our CD data, introducing the R91C mutation resulted in ~9 °C and ~15 °C decreases T_m_ in the absence and the presence of DTT, respectively (Table 1). If an SS bond can be formed by C91, we would expect an increase in stability. However, side chains of cysteine residues at the 91st position are located on the opposite sides of the coiled coil and cannot form the SS bond (Figure S1). Thus, the difference between T_m_ in the presence and the absence of DTT can be attributed to the disulfide bond formed by Cys in the N-termini of the designed fragments and not by C91. A disrupted coiled coil in Tpm3.12_65–155_R91C contributes to the reduced affinity of Tpm for actin shown previously [32].

Studies on the thermostability of the full-length Tpm3.12-R91P showed that the substitution caused a conformational change, which resulted in a loss of the N-terminal calorimetric domain [30]. In this work, we showed that the substitution severely affected the region near the substitution. Given the destructive effect of Pro on the α-helix formation [33], the significant loss of the α-helical structure resulting in a dramatic loss of stability of Tpm3.12_65–155_R91P is not surprising. Along similar lines, the R91P mutation instigated the disruption of coiled-coil structure as shown by the difference of 32 °C in T_m_ in the absence of DTT. T_m_ could not be determined in the presence of DTT as the Tpm3.12_65–155_R91P had already begun unfolding at 0 °C (Table 1, Figure 3). The presence of DTT prevented the formation of an SS bond at the N-terminal sequence, thereby attributing to the drastic difference in the stability of the Tpm3.12_65–155_R91P fragment. Tpm3.12_65–155_R91P loses its coiled-coil structure significantly as indicated by the increase in the disorder and loss of helical content (Figure 4).

Surprisingly, in the 200 ns MDS run, the substitution of R91 with P showed no significant changes in the coiled-coil structure of Tpm3.12_65–155_R91P. Conventional MDS for time frames of 200 ns and similar have been useful in determining the effects of mutations on protein structural behavior and protein–protein interactions [34]. However, it still remains a challenge to show an experimentally-determined disruptive and slow dissociation of protein–protein interactions due to time frames ranging from micro to milliseconds or even longer [35,36]. Due to the limitations of conventional MDS and the use of shorter timeframes, specific disruptive effects of the Tpm3.12_65–155_R91P and Tpm1.1_65–155_I92T mutation on coiled coil destabilization seen in the CD experiments remain elusive.

The mutations I92T and R91P result in the loss of the local coiled-coil structure in the region of the actin-binding period 3. At the body temperature (~37 °C), this region becomes unfolded; this should drastically affect the binding of Tpm to F-actin. Two other mutations, V95A and R91C, cause partial unfolding in this region at the body temperature, the interactions between Tpm and F-actin should also be affected, although to a lesser extent. Moreover, Tpm has a multidomain structure with several cooperatively melting domains, and the unfolding of one domain has allosteric effects on the stability of the subsequent domains [37].

## 4. Materials and Methods

### 4.1. Construction and Cloning of TPM1 and TPM3 cDNA Fragments

cDNA encoding fragments of Tpm1.1 and Tpm3.12 comprising amino acid residues 64–154 or 65–155 were excised from *TPM1* and *TPM3* cDNAs encoding human skeletal tropomyosin isoforms using PCR amplification.

The *TPM1* cDNA sequence was amplified by PCR with forward:

5′TAGTACATATGTGCGGCGGCCTCAAAGATGCCCAGGAG3′ and reverse

5′TAGATGGATCCTTAAATGTGCTTGGCCTCTTTC3′ primers.

The *TPM3* cDNA sequence was amplified with forward:

5′TAGTACATATGTGCGGCGGCCTGAAAGACGCTCAGGAAAAACTG 3′ and reverse 5′TAGATGGATCCTTAGATGTGTTTAGCTTCTTTCAGC3′ primers. The *Nde*I restriction site and the sequence encoding CysGlyGly upstream of the sequences encoding Tpm1.1 and Tpm3.12 were added to the 5′-end of the fragments. A *Bam*HI restriction site and a TAA translation termination codon were introduced at the 3′-end of the amplified fragments. DNA fragments were amplified by PCR using Taq DNA polymerase (New England BioLabs, Inc., Ipswich, MA, USA) and the appropriate PCR buffer at 10 × concentration with 17.5 mM MgCl_2_. The reaction mixture (50 μL) contained 0.15 mM dNTPs, 1 × Taq DNA polymerase buffer, 30 pmoles of each primer, 1U polymerase, and 80 ng of template plasmid. The first amplification was preceded by 4 min incubation at 94 °C and then DNA polymerase was added. The amplification parameters were as follows: denaturation at 94 °C for 30 s and extension at 72 °C for 25 s. Annealing was conducted for 15 s at 48 °C for *TPM1* and at 50.5 °C for *TPM3*. The number of amplification cycles was set to 20. The procedure was completed by 5 min incubation at 72 °C. The PCR fragments were digested with *Nde*I and *Bam*HI. The obtained sequences were placed under the transcriptional control of the T7 promoter of T7 bacteriophage in the pET15b expression vector. For this purpose, *TPM1* and *TPM3* sequences coding amino acid residues 64–154 and 65–165 were cloned into the pET15b backbone between the *Nde*I and *Bam*HI restriction sites. The plasmids were amplified in the DH5α strain (Stratagene, La Jolla, CA USA) according to the method of the manufacturer. The validity of the cDNA sequences introduced into the pET15b plasmid was confirmed by DNA sequencing.

### 4.2. Site-Directed Mutagenesis

Fragments of *TPM1* and *TPM3* cloned into the pET15b vector were used as templates. Cardiomyopathy-related mutations I92T and V95A were introduced into the *TPM1* cDNA. CFTD-causing mutations R91C and R91P were introduced into the *TPM3*. The mutations were inserted using a PCR-based oligonucleotide-directed mutagenesis kit (Agilent Technologies, Santa Clara, CA, USA). The following oligonucleotides were used:

I92T: forward 5′CATCTCTGAACAGACGCACCCAGCTGGTTGAGG3′

and reverse 3′GTAGAGACTTGTCTGCGTGGGTCGACCAACTCC5′;

V95A: forward 5′ACGCATCCAGCTGGCTGAGGAGGAGTTGG3′

and reverse 3′TGCGTAGGTCGACCGACTCCTTCAACC5′

R91C: forward 5′GAAGTTGCTTCTCTGAACTGTCGTATCCAGCTGGTTG3′

and reverse 3′CTTCAACGAAGAGACTTGACAGCATAGGTCGACCAAC5′;

R91P: forward 5′GAAGTTGCTTCTCTGAACCCTCGTATCCAGCTGGTTG3′

and reverse 3′CTTCAACGAAGAGACTTGGGAGCATAGGTCGACCAAC5′.

The changed codons are underlined. The oligonucleotides were synthesized and HPLC was purified by the Laboratory of DNA Sequencing and Oligonucleotide Synthesis, Institute of Biochemistry and Biophysics (Warsaw, Poland). The PCR reaction was performed according to the method supplied by the manufacturer (https://www.agilent.com/cs/library/usermanuals/public/200523.pdf, accessed on 15 November 2021). The amplification reaction involved initial DNA melting (95 °C, 30 s), then 11 cycles of melting (95 °C, 30 s), and primers annealing (55 °C, 1 min) and extending (68 °C, 6 min). The parental DNA template was digested with the DpnI restriction enzyme and PCR products were transformed into XL-1 supercompetent cells, DNA was isolated (GeneMATRIX Plasmid Miniprep DNA Purification Kit, EURx), and the substitutions were verified by DNA sequencing in the Laboratory mentioned above.

### 4.3. Peptide Expression and Purification

Wild-type and mutant Tpm1.1_64–154_ and Tpm3.12_65–155_ were expressed in *E.coli* BL21 (DE3) competent cells (Novagen Inc. Madison, WI, USA). The cells were transformed with wild-type and mutant plasmids *TPM1* and *TPM3* by 30-sec heat shock at 42 °C according to the protocol of the manufacturer. Transformed cells were grown overnight in Luria–Bertani (LB) broth supplemented with 0.05 mg/mL ampicillin, at 37 °C, without shaking. Peptide production was induced by 0.4 mM IPTG at culture OD_550_ between 0.2 and 0.4. The culture was grown with shaking 270 rpm at 37 °C until OD_550_ reached 1.0. Cells were pelleted by 10 min centrifugation in a GSA Sorval rotor at 11,000 rpm, 4 °C. To release the peptides, pellets collected from 600 mL of culture were suspended in 12 mL of lysis buffer (50 mM Tris-HCl, pH 7.5, 10 mM EDTA, 20% saccharose, 0.5 mg/mL lysozyme), homogenized in Dounce homogenizer, incubated for 1 h on ice, and then for 10 min at −80 °C. After thawing, NaCl was added to a 0.2 M final concentration, and the extract was frozen for 10 min at −80 °C. Finally, NaCl concentration was increased to 1 M, and the solution was subject to two cycles of sonication using the Sonicator (Bandelin Sonoplus HD2070—BANDELIN electronic GmBH & Co. Berlin, Germany) for 3 min at 70% maximal power. Cell debris was pelleted in Beckman rotor 70Ti for 1 h, at 40,000 rpm, 4 °C. Supernatants were collected and incubated in a water bath at 100 °C for 4 min. The denatured proteins were pelleted in a 70Ti rotor, at 30,000 rpm, at 4 °C for 30 min. Supernatants were dialyzed overnight to A-buffer (50 mM phosphate buffer, pH 8.0, 0.3 M NaCl, 10 mM imidazole, 0.02% NaN_3_). The peptides were purified by affinity chromatography. The protein solution was loaded on His-Select Nickel Affinity Resin (Sigma-Aldrich Corp. St Louis, MO, USA) column equilibrated with A-buffer and eluted with B-buffer (50 mM phosphate buffer pH 8.0, 0.3 M NaCl, 250 mM imidazole, 0.02 % NaN_3_). Fractions from 2/3 of the elution peak were collected and dialyzed exhaustively against 20 mM Tris pH 7.5, 0.1 M NaCl, 10 mM EDTA, 0.02% NaN_3_. His-tag was removed from the peptides by the Thrombin Cleavage Capture Kit (Novagen Inc., Madison, WI, USA). His-tagged Tpm peptides were incubated overnight at room temperature with biotinylated thrombin (0.02 U/1 mg of protein). After digestion, thrombin was removed by incubation for 30 min at room temperature with streptavidin-agarose resin, followed by centrifugation in filter columns. Protein concentration was measured with the micro-biuret method [38]. Peptides were dialyzed to 50 mM (NH_4_)HCO_3_, lyophilized, and stored at −80 °C. The identity of the peptides was confirmed by mass spectrometry (Liquid Chromatography and Mass Spectrometry Laboratory, Faculty of Biology, University of Warsaw, Poland). The obtained molecular weights (MW) corresponded to the theoretical MWs of dimers with oxidized Cys residues calculated in Expasy (https://web.expasy.org/compute_pi/, accessed on 15 November 2021) based on the sequences of the peptides.

### 4.4. Peptide Concentration Determination

Protein concentration was measured with the micro-biuret method [38] or/and with the Pierce^TM^ BCA protein assay kit (Thermo Fisher Scientific, Waltham, MA, USA). BCA uses the combination of the biuret reaction and the colorimetric detection of cuprous cations (Cu^+^), which are influenced by the presence of copper-reducing amino acids cysteine, tyrosine, and tryptophan. Due to the lack of tyrosine and tryptophan residues in the Tpm fragments, other standard methods for protein concentration determination could not be used.

### 4.5. Circular Dichroism Measurements

Samples for CD experiments were dissolved in 10 mM sodium phosphate, 100 mM NaCl, pH 7.0 to a final concentration of ~0.1 mg/mL. Tpm3.12_65–155_ and Tpm3.12_65–155_R91P fragments were resuspended in appropriate volumes of 100 mM Tris pH 8.0, 0.1 M PMSF. The samples were acidified to a final concentration of 0.2 M HCl using 1 M HCl. The samples were purified using a Sep-Pak C18 cartridge (Waters, Milford, CA, USA) and were eluted using a solution of 0.1% trifluoroacetic acid (TFA) containing 60% acetonitrile. The eluted samples were lyophilized to get rid of acetonitrile. Any impurities, if present, were removed by the Vydac 218TP C18 HPLC column using a linear gradient of acetonitrile, 1% per minute, in the presence of 0.1% TFA. An HPLC fraction containing the majority of the Tpm3.12 peak was collected in a glass vial and lyophilized. No additional peaks were found during HPLC, indicating the absence of any significant impurities. Lyophilized fractions were pooled and resuspended using minimal volumes of autoclaved HPLC water. The pooled fractions were lyophilized.

After the lyophilized Tpm fragments were resuspended, we noticed that not all fragments were soluble. In order to improve the solubility of the fragments, we refolded them by dialysis in the presence of urea. Tpm3.12_65–155_ was resuspended in 20 mM Tris pH 8.0, 8 M urea, and 100 mM DTT. The resuspended samples were dialyzed against 20 mM Tris pH 8.0 with concentrations of urea gradually reduced to 0 M in the following order: 8 M–6 M–5 M–4 M–3.5 M–3 M–2.5 M–2.0 M–1.5 M–1 M–0.5 M–0 M, with a 12h duration between each step change. At 4 M urea, the concentration of DTT was changed to 1 mM and 100 mM NaCl was introduced into the dialysis buffer. Dialyzed soluble Tpm3.12_65–155_ were stored at −80 °C until further use.

CD spectra measurements were performed using an Aviv model 400 spectropolarimeter (Lakewood, NJ, USA) in 1 mm quartz cuvettes. Thermal denaturation measurements were obtained by monitoring the ellipticity signal at 222nm as a function of temperature. For each unfolding curve, a melting temperature (T_m_) was determined as a maximum of the first derivative using the Aviv instrument control program—Aviv 420 cdsxm (Lakewood, NJ, USA). Melting data for Tpm fragments were normalized using the following equation:

$$z_{i}=\frac{x_{i}-\min\left(x\right)}{\max\left(x\right)-\min\left(x\right)}$$

where, x_i_ represents the CD signal at a given temperature, min(x) represents the lowest CD signal, and max(x) represents the highest CD signal where there is maximal folding. Thermal denaturation profiles were normalized and plotted against temperature using SigmaPlot 12 (Systat Software, San Jose, CA, USA).

CD spectra for the purified fragments, additionally purified using HPLC, were compared with fragments without the additional purification and no difference in spectra was observed. Therefore, purification and refolding procedures were not repeated for other Tpm fragments.

### 4.6. Molecular Dynamics Simulations

Protein structure building, editing, and visualization were performed with UCSF Chimera [39]. To build a starting structure for Tpm1.1_64–154_, the X-ray crystal structure of cardiac α-tropomyosin from *Sus scrofa* (PDB ID 1C1G) was used as a template to create PDB files for Tpm1.1_64–154_ and Tpm3.12_65–155_. The crystal structure was edited by deleting M1 to A64 and A155 to I284 amino acid residues from chains B and D, and the remaining residues were changed according to the Tpm1.1 sequence (res. 64–154). The sequence *GSHMCGG* was added to the N-terminus of the fragment. A starting structure for Tpm3.12_65–155_ was created by changing four residues in the Tpm1.1_64–154_ structure.

Structures were neutralized with Na^+^ or Cl^−^ ions and solvated in TIP3P water [40] with at least 10 Å between the structure and the edge of the solvation box. Energy minimizations were performed by the *sander* protocol in Amber18 [41] with 2500 cycles of the steepest descent method followed by 2500 cycles of the conjugate gradient method. Production runs were performed by the GPU-accelerated *pmemd* implementation of the *sander* protocol with a 1 fs time-step, using the ff14SB force field [42], and periodic boundary conditions. The temperature was controlled by a Langevin thermostat with a collision frequency of 3 ps^−1^. Covalent bonds to hydrogen were constrained by SHAKE. The structures were minimized before being subjected to a 200 ns simulation run at 298 K. PDB files were generated from the 200 ns simulated structures and were visualized to identify the first and last α-helix-forming residues for Tpm fragments, Tpm1.1_64–154_ and Tpm3.12_65–155_. For each Tpm fragment (wild-type and mutated), the distance (end-to-end distance) between these residues was determined over the course of 200 ns for both chains in the coiled coils using the corresponding trajectory and topology files. The same files were used to create movies for the 200 ns simulation run using UCSF Chimera.

### 4.7. Statistical Analysis

Statistical analysis for CD data was performed using paired (to compare wild-type with the mutants) and unpaired t-test (to compare results with and without DTT). Obtained *p*-values were less than 0.0003 (most being even less than 0.00001) demonstrating the difference is extremely statistically significant.

## 5. Conclusions

We can conclude that specific conformations adopted by period 3 of Tpm isoforms 3.12 and 1.1 define many Tpm interactions and, consequently, the performance of the thin filament. Disease-causing mutations disrupt the structure of the Tpm coiled coil; however, local destabilization caused by some mutations may not be detected in full-length Tpms. Using designed Tpm fragments allowed us to determine even slight changes in the stability of the actin-binding domain 3 caused by the mutations.

## Figures and Tables

**Table 1:** Effect of the mutations on melting temperature (T_m_) of Tpm1.1_64–154_ and Tpm3.12_65–155_ under non-reducing and reducing conditions. Melting temperatures were determined from melting curves measured in 10 mM Na-phosphate, 100 mM NaCl, pH 7.0, under reducing (0.5 mM DTT) and non-reducing (no DTT) conditions. ND: not determined. The numbers are mean values from three measurements ± SE.

Tpm	Melting Temperature (T_m_), °C	
	No DTT	DTT
Tpm1.1_64–154_	46.1 ± 0.2	23.7 ± 0.7
Tpm1.1_64–154_I92T	28.2 ± 0.1	5.6 ± 0.5
Tpm1.1_64–154_V95A	42.2 ± 0.5	21.7 ± 0.3
Tpm 3.12_65–155_	60.9 ± 0.2	45.8 ± 0.9
Tpm 3.12_65–155_R91C	51.5 ± 0.7	31.3 ± 0.2
Tpm3.12_65–155_R91P	28.9 ± 0.1	ND

## Data Availability

The data presented in this study are available from the authors.

## Supplementary Materials

The following are available online, Figure S1: Effects of mutations on Tpm1.1_64–154_ and Tpm3.12_65–155_ structures. Figure S2: Effects of mutations on the flexibility of Tpm1.1_64–154_ and Tpm3.12_65–155_. Distance between Cα atoms of the first and last α helical residues for chains A and B of Tpm fragments, measured over the course of a 200 ns MDS run. Figure S3: End-to-end distance between Cα atoms of the first and last α-helical residues for Chains A and B of Tpm fragments Tpm1.1_64–154_ (A) and Tpm1.1_64–154_I92T (B) with changed side chain positions, measured over the course of a 200 ns MDS run. Table S1: The average end-to-end distances of Tpm fragments.

## Sample Availability

Samples of the cDNA of tropomyosin peptides are available from the authors.
