# Supplementary material for: Structural Effects of Disease-Related Mutations in Actin-Binding Period 3 of Tropomyosin
Source: Molecules. 2021 Nov 19;26(22):6980. doi: 10.3390/molecules26226980 (PMC8622905; doi:10.3390/molecules26226980)
Supplement: Supplementary file 1 [file molecules-26-06980-s001.zip › molecules-1439275-SM.pdf]

molecules

MDPI

Supplementary Materials

Structural Effects of Disease-Related Mutations in Actin-Binding Period 3 of Tropomyosin

Balaganesh Kuruba [1], Marta Kaczmarek [2], Małgorzata Kęsik-Brodacka [3], Magdalena Fojutowska [2], Małgorzata Śliwinska [2], Alla S. Kostyukova [1] and Joanna Moraczewska [2,*]

1 Gene and Linda Voiland School of Chemical Engineering and Bioengineering, Washington State University, Pullman, WA 99163, USA; balaganesh.kuruba@wsu.edu(R.K.); alla.kostyukova@wsu.edu(A.S.K.)
2 Department of Biochemistry and Cell Biology, Faculty of Biological Sciences, Kazimierz Wielki University, 85-671 Bydgoszcz, Poland; marta92@ukw.edu.pl(M.K.); magdalena.fojutowska@ukw.edu.pl(M.F.); gosia.sl@ukw.edu.pl(M.Ś.)
3 National Medicines Institute, 00-725 Warsaw, Poland; m.kesik@nil.gov.pl
* Correspondence: joanna.moraczewska@ukw.edu.pl; Tel.:+48-52-52-325-9219

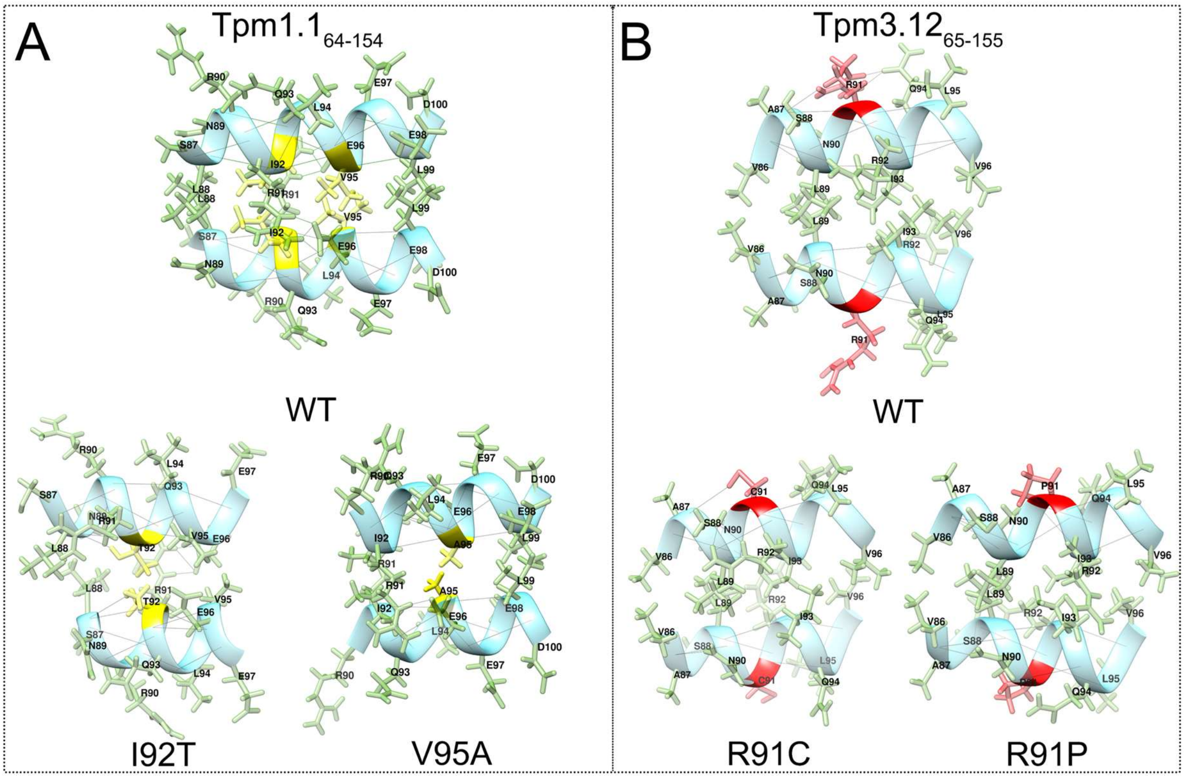

Figure S1. Effects of mutations on Tpm1.1$_{64–154}$ and Tpm3.12$_{65–155}$ structures.

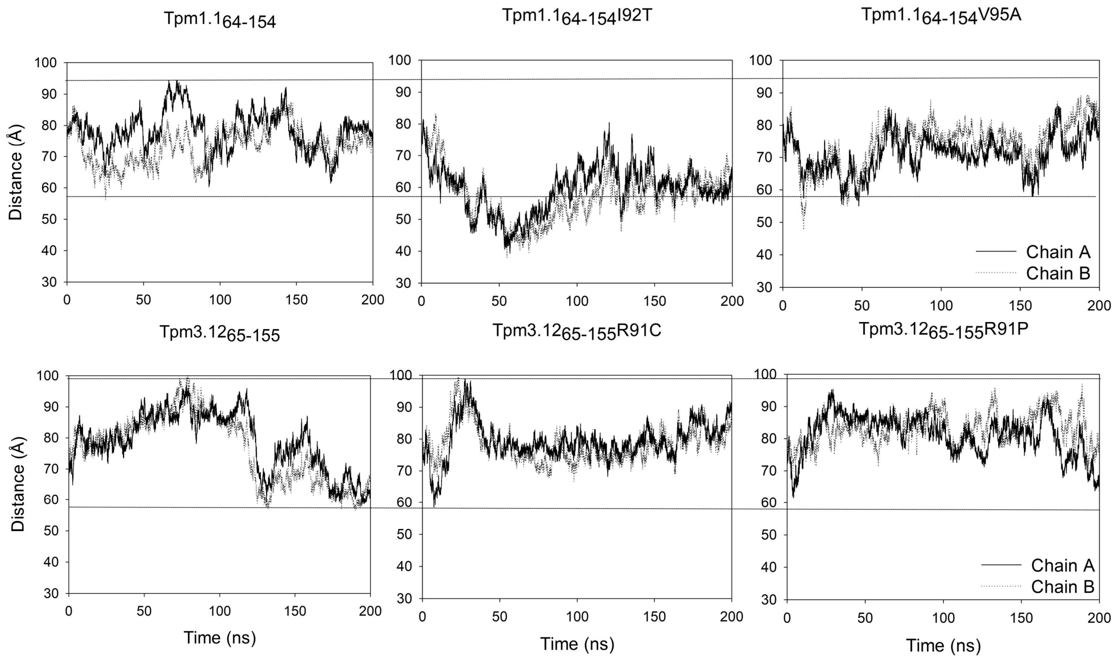

Figure S2. Effects of mutations on the flexibility of Tpm1.1$_{64–154}$ and Tpm3.12$_{65–155}$. Distance between Cα atoms of the first and last α helical residues for chains A and B of Tpm fragments, measured over the course of a 200 ns MDS run.

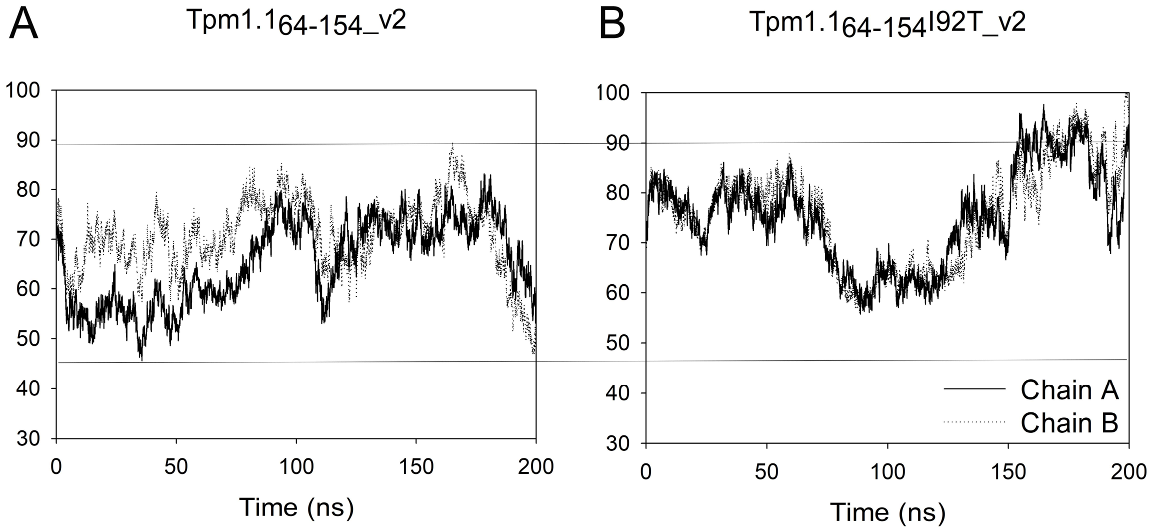

Figure S3. End-to-end distance between Cα atoms of the first and last α-helical residues for Chains A and B of Tpm fragments Tpm1.1$_{64–154}$ (**A**) and Tpm1.1$_{64–154}$I92T (**B**) with changed side chain positions, measured over the course of a 200 ns MDS run.

Table S1. The average end-to-end distances of Tpm fragments. Numbers are means ± standard deviations of the distances observed for 4,000 frames during a 200 ns MDS run. The distances are in Angstroms (Å).

**Tpm1.1$_{64-154}$**		**Tpm1.1$_{64-154}$I92T**		**Tpm1.1$_{64-154}$V95A**	
**75.8 ± 4.9**		**58.4 ± 7.2**		**72.8 ± 5.9**	
Chain A	*Chain B*	*Chain A*	*Chain B*	*Chain A*	*Chain B*
78.2 ± 6.2	73.4 ± 5.7	59.5 ± 7.4	57.3 ± 8.1	71.0 ± 5.6	74.7 ± 6.9
**Tpm3.12$_{65-155}$**		**Tpm3.12$_{65-155}$R91P**		**Tpm3.12$_{65-155}$R91C**	
77.8 ± 9.9		82.2 ± 4.6		78.9 ± 5.3	
Chain A	*Chain B*	*Chain A*	*Chain B*	*Chain A*	*Chain B*
78.9 ± 9.3	76.7 ± 11.1	81.3 ± 6.2	83.1 ± 5.0	79.4 ± 5.8	78.3 ± 5.7
